# Knowledge, attitudes, and practices for the use of seasonal influenza vaccination, healthcare workers, Costa Rica

**DOI:** 10.3855/jidc.14381

**Published:** 2021-07-31

**Authors:** Zachary J Madewell, Rafael Chacón-Fuentes, Xiomara Badilla-Vargas, Catalina Ramirez, Maria-Renee Ortiz, Juan-Pablo Alvis-Estrada, Jorge Jara

**Affiliations:** 1 Centro de Estudios en Salud, Universidad del Valle de Guatemala, Guatemala City, Guatemala; 2 Caja Costarricense del Seguro Social, San José, Costa Rica

**Keywords:** Central America, immunization, vaccination coverage, health personnel, influenza vaccines

## Abstract

**Introduction:**

Annual seasonal influenza vaccination in healthcare workers prevents nosocomial transmission to patients, coworkers, and visitors, and reduces absenteeism. This study aimed to describe knowledge, attitudes, and practices (KAP) of seasonal influenza vaccine among public healthcare workers attending patients in Costa Rica.

**Methodology:**

We conducted a cross-sectional survey of healthcare personnel attending patients in public hospitals in 2017–2018. Frequency distributions of demographics, vaccination KAP, sources of information, clinical manifestations and reasons for non-vaccination were reported. Logistic regression was used to analyze associations between exposures of interest (demographics, sources of information, knowledge, attitudes towards vaccination) and self-reported influenza vaccination.

**Results:**

We surveyed 747 healthcare workers in 2017–2018. Of 706 participants who knew their vaccination status, 55.7% were vaccinated for seasonal influenza. Only 20.7% of participants knew the influenza vaccine was an inactivated virus, and 94.6% believed the vaccine causes flu-like symptoms. Factors associated with current influenza vaccination were vaccination in previous year (aOR: 8.13; 95% CI: 5.65–11.71) and believed influenza vaccination may be harmful (aOR: 0.62; 95% CI: 0.44–0.89). Reasons for non-vaccination included fear of adverse effects and access limitations.

**Conclusions:**

Suboptimal influenza vaccination among healthcare workers may be attributed to misconceptions about the vaccine and limited engagement strategies focusing on healthcare workers. Appropriate interventions are needed to increase healthcare worker vaccination rates and improve their knowledge and beneficence, which would improve patient safety in hospitals.

## Introduction

Influenza is a highly infectious respiratory disease that is usually mild or moderate at presentation, but may cause serious pulmonary, neurological and cardiac complications or death, particularly among risk groups [1]. Influenza viruses are spread via direct, indirect, or close contact with infected people via respiratory droplets or saliva [1]. About half of influenza infections are symptomatic, but asymptomatic individuals may spread the virus for 24 hours before symptom onset [2]. Worldwide, annual influenza epidemics are estimated to result in 500–800 million infections, 3–5 million cases of severe illness, and 290,000–650,000 deaths [3]. This study focuses on Costa Rica where influenza incidence, hospitalizations, and deaths were 412.6 (95% CI: 277.5–581.3), 38.5 (95% CI: 12.2–109.9), and 0.6 (95% CI: 0.4–1.0) per 100,000, respectively, in 2017 [4]. In 2017, Costa Rica reported 1.5 million upper acute respiratory infections, 52,000 influenza-like illnesses, and 4,200 severe acute respiratory infections [5].

Vaccination is the most effective measure to prevent new infections and reduce seasonal influenza-associated morbidity and mortality [6,7]. Vaccine effectiveness against illness ranged from 10–60% from 2004–2020 [8]. World Health Organization and Advisory Committee on Immunization Practices recommend healthcare workers get vaccinated for seasonal influenza annually [6,7]. Compared to the general population, healthcare workers are at greater risk of influenza infection and up to one-quarter contract influenza annually [9]. Annual influenza vaccination in healthcare workers reduces absenteeism, allowing healthcare services to continue during influenza outbreaks, and prevents nosocomial transmission to hospital patients, coworkers, and visitors [6,7]. Healthcare-related influenza transmission has the potential to trigger large outbreaks within healthcare facilities, which may temporarily close entire healthcare facilities [10,11]. Despite influenza vaccination recommendations, coverage among healthcare workers remains low worldwide [12] and many healthcare workers continue working while sick favoring the spread of influenza transmission [13]. Factors underlying vaccine hesitancy among healthcare workers are no different from the general population and include concerns regarding vaccine safety, fear of adverse effects, poor accessibility, and doubts about vaccine effectiveness [14,15]. Healthcare workers with positive views on benefits of influenza vaccination are more likely to recommend vaccination to their patients [16]. In Central American countries, influenza epidemics usually begin in May (± two months) and last four months [17]. High risk groups are vaccinated for seasonal influenza during annual vaccination campaigns from April-June [18]. However, the seasonal influenza vaccine is not used uniformly across Central American countries, ranging from 10% coverage among healthcare workers in Belize to 100% in Honduras in 2017 [19]. Understanding attitudes and behaviors of healthcare workers towards vaccination may lead to strategies to improve coverage. This study therefore aimed to describe knowledge, attitudes, and practices (KAP) of seasonal influenza vaccination in Costa Rica.

## Methodology

### Study design

This study was a cross-sectional KAP survey regarding seasonal influenza vaccination among healthcare workers who attended patients in public hospitals of the Costa Rican Social Security Fund (CCSS).

### Study Setting

Costa Rica has an area of 51,100 km^2^, conformed by 7 provinces and 82 cantons [20]. It has a population of approximately 5,100,000 inhabitants, population density of 99 people per square kilometer, and 80.8% resided in urban areas [20]. The life expectancy is 79.2 years (female: 82.0 years; male: 76.5 years) and mortality rate is 4.9 deaths per 1,000 population [20]. Costa Rica’s national public health expenditure was 5.6% of its GDP in 2016, slightly higher than the 3.7% for all of Latin America and the Caribbean [21]. CCSS has provided public healthcare services in Costa Rica since 1973. Public hospitals cover 87% of Costa Rica’s population [22]. CCSS introduced influenza vaccination in 2004 to target groups: children 6–23 months of age, persons with chronic diseases; adults >60 years of age; pregnant women at any gestational age; and healthcare personnel. Influenza vaccines are available free-of-charge for risk groups at CCSS healthcare facilities nationwide during vaccination campaigns.

### Questionnaire

A questionnaire was adapted from the U.S. Centers for Disease Control and Prevention influenza survey [23]. The questionnaire was modified following an evaluation of technical detail and cultural appropriateness by an anthropologist and personnel from the Center for Strategic Development and Information on Health and Social Security of CCSS and Institutional Review Board of Universidad del Valle de Guatemala (UVG). The questionnaire was pilot-tested with a group of healthcare workers (medical doctors, nurses, laboratory personnel) at CCSS in May 2017, four weeks before study implementation. Minimal modifications were made to the questionnaire following feedback provided by the participants. The finalized questionnaire included demographics, knowledge and attitudes of influenza vaccination, vaccination status, sources of information of influenza vaccination, clinical manifestations following vaccination, and reasons for non-vaccination.

A close-ended survey was conducted to healthcare personnel attending patients in hospitals between 26 June and 11 August 2017 and between 17 September and 26 October 2018, antedating the peaks of influenza A and B epidemics in Costa Rica [24]. Surveys in 2017 were temporarily suspended in order to complete new requirements requested by the National Health Research Council of Costa Rica. Trained healthcare professionals surveyed participants by interviews in CCSS hospitals and collected data with tablets using the Research Data Management Center application (Open Data Kit ODK JAVA).

### Study population

The lowest administrative vaccination coverage for influenza (PAHO) was used to calculate the sample size of healthcare workers: 38% [19]. The number of healthcare workers listed for CCSS hospitals (21,550 people) was used as the reference population [25]. A design effect of two was used, corresponding to the two stages of sampling described below. Applying 5% accuracy, 95% confidence interval, and 10% replacement rate, a sample size of 860 healthcare workers was calculated (Supplementary File 1).

**Figure 1.**
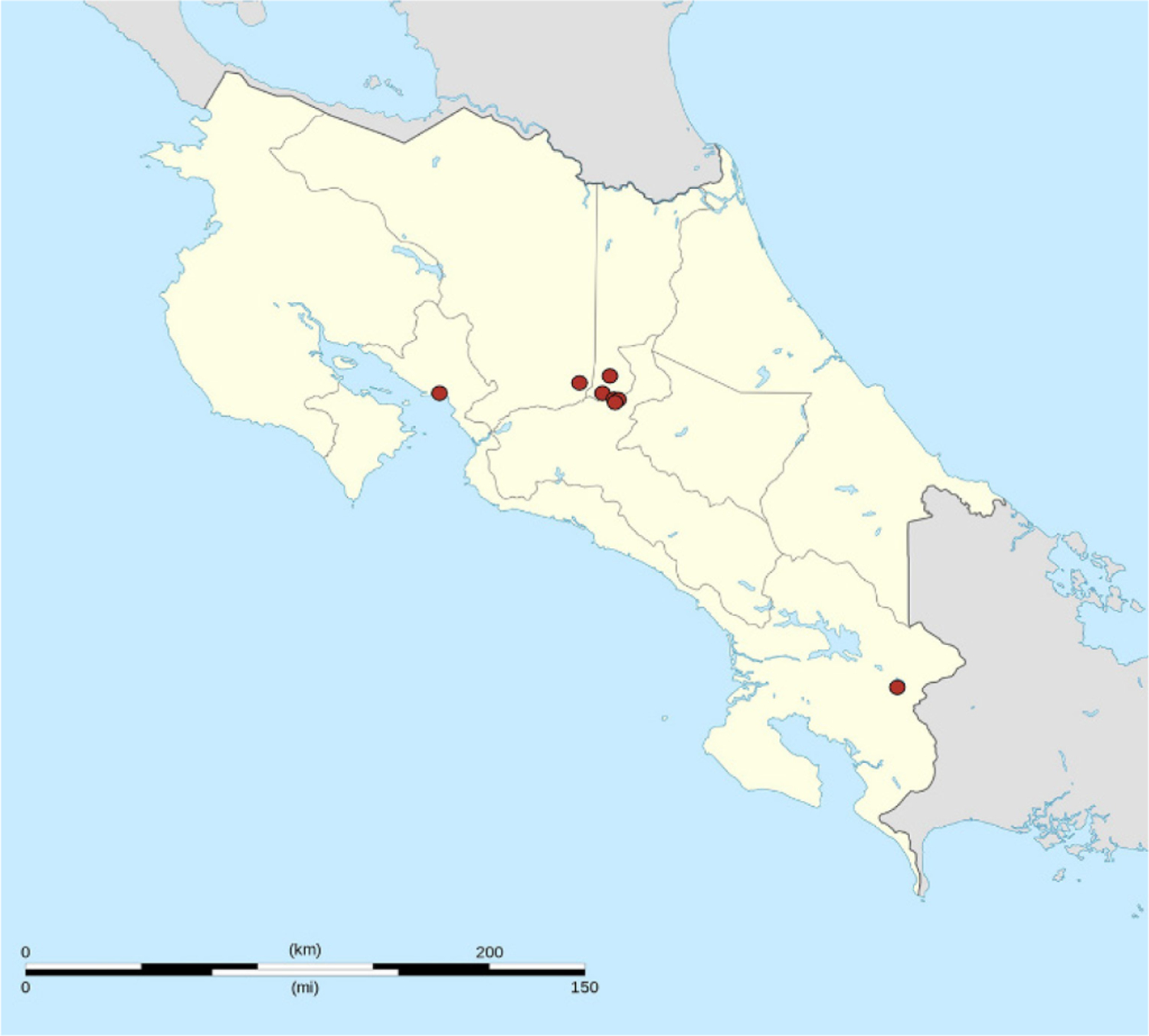
Locations of healthcare facilities from Costa Rican Social Security Fund, study of knowledge, attitudes and practices of seasonal influenza vaccination, healthcare workers, Costa Rica, 2017–2018. Source: Costa Rica location map; by user Eric Gaba; licensed under CC BY 3.0 via Wikimedia Commons, https://commons.wikimedia.org/wiki/File:Costa_Rica_location_map.svg.

Two-stage stratified cluster sampling was used to select samples of healthcare workers who worked in hospitals of CCSS. Stratification was based on hospital health network location (Eastern, South, Northwest). In stage one, we identified conglomerates (hospitals) in each stratum (health network) by probability proportional to the number of healthcare workers attending patients in each healthcare facility. In stage two, we identified healthcare workers in each selected conglomerate by simple random sampling within each group of healthcare professionals. Healthcare facilities were located in five of the seven departments of Costa Rica (Figure 1). Healthcare workers ages ≥ 18 years attending patients in CCSS establishments were invited to participate. Healthcare workers were doctors (general practitioners or medical specialists), nurses (auxiliary or professional), and other healthcare workers in direct contact with patients (e.g., dentists, psychologists, social workers, radiology technicians, laboratory staff, cleaning staff, customer service staff, others). Excluded participants were administrative or support staff who did not attend patients.

### Statistical analysis

Frequency distributions of demographics (age, gender, marital status, profession, years in profession, works in multiple healthcare facilities, current and previous year self-reported vaccination status) were reported. Frequency distributions and 95% confidence intervals (CI) for knowledge and attitudes of influenza virus, transmission, and vaccination; sources of information for vaccination; clinical manifestations seven days after vaccination; and reasons for non-vaccination were also reported. Results were reported for all participants and stratified by survey year as KAP regarding influenza vaccination and the environment (e.g., vaccine efficacy, communication strategies, intensity of influenza season, surveillance activities) may have differed between survey years. Pearson Chi-square tests were used to evaluate associations between demographics, knowledge, attitudes, sources of information, clinical manifestations, and reasons for non-vaccination, and survey year. Logistic regression was used to analyze associations between demographics, information sources, knowledge, and attitudes about the influenza vaccine, and self-reported influenza vaccination for 1) all participants, 2) participants in 2017, and 3) participants in 2018. These analyses excluded people who did not know or did not provide their vaccination status and those who did not respond to knowledge or attitude questions. Statistical significance was evaluated through the Wald Chi-square test. Variables found to be significant at *p* < 0.10 from unadjusted analyses were included in step-wise multivariable logistic regression models to evaluate associations with influenza vaccination status. Variables associated with vaccination at p<0.05 were retained in the final model. Tolerance values were used to assess collinearity among all independent variables and Hosmer-Lemeshow to assess the final adjusted model’s goodness-of-fit. SAS V.9.4 (SAS Institute, Inc., Cary, North Carolina) was used for all analyses.

### Ethics statement

This study was approved by the Research Ethics Committee of UVG (Protocol number 156-11-2016) and Center for Strategic Development and Information on Health and Social Security of CCSS (study code AB-1513-17). It was registered with the National Health Research Council of Costa Rica. Written informed consent was obtained for all participants.

## Results

### Sample characteristics

A total of 747 healthcare workers who attended patients in nine CCSS hospitals were surveyed: 553 in 2017 and 194 in 2018. The median age of all participants was 37 years (interquartile range: 31–48 years) and median years in profession was 11 years (interquartile range: 7–20 years) (Table 1). Of all participants, 59.6% were female, 52.3% were married, and 41.6% were nursing professionals or assistants. Of 196 physicians, 68.9% had medical specialization.

### Knowledge and attitudes of influenza vaccination

Almost all participants believed healthcare workers should be vaccinated for seasonal influenza annually (96.1%), but 38.1% did not recognize influenza may be transmitted from birds or pigs to people (Table 2). Furthermore, only 20.7% of healthcare workers knew the seasonal influenza vaccine offered by CCSS was composed of inactivated viruses and 94.6% believed the vaccine causes flu-like symptoms. Compared to healthcare workers in 2017, a greater proportion of healthcare workers in 2018 knew that someone may become infected with influenza multiple times, knew influenza may be transmitted via contaminated hands, and indicated they would get vaccinated for influenza if offered the vaccine at work, whereas fewer believed vaccination may be harmful (*p*-values ≤ 0.03) (Supplementary Table 1).

### Sources of information

Of 747 healthcare workers, 466 learned about the influenza vaccine from mass media (62.5%) and 439 from informal information (e.g., pamphlets, posters) at the healthcare facility (58.8%) (Table 3). A greater proportion of healthcare workers cited trainings at healthcare facilities as sources of information about influenza vaccination in 2018 compared to 2017 and fewer cited mass media, friends, and family (*p*-values ≤ 0.01) (Supplementary Table 2).

### Influenza vaccination

**Figure 2.**
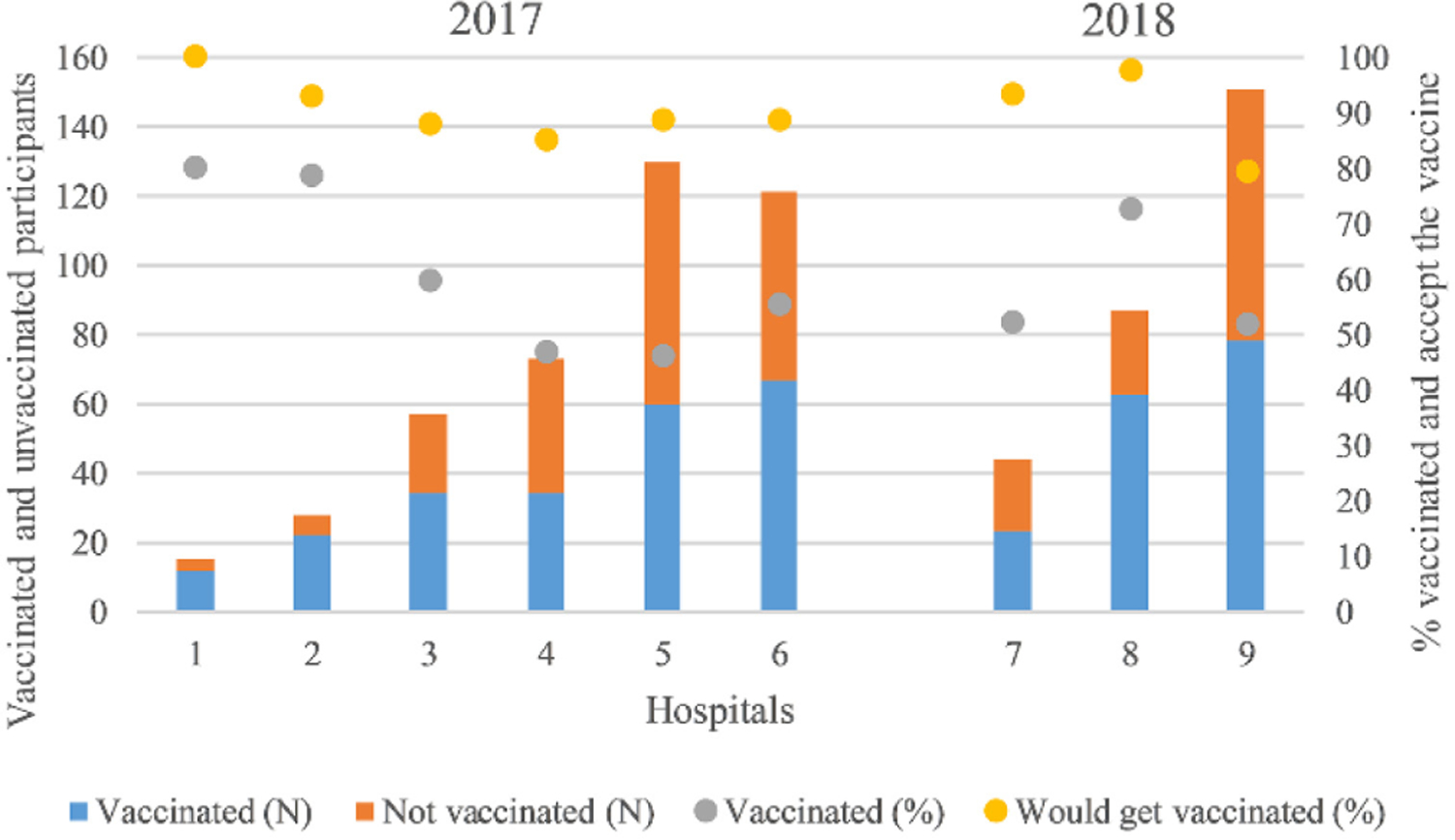
Seasonal influenza vaccination coverage among 553 and 194 healthcare workers in 2017 and 2018, respectively, and proportion who would get vaccinated if offered the vaccine at work by hospital, Costa Rica, 2017–2018.

In 2017, 279 of 516 healthcare workers self-reported vaccination for seasonal influenza (54.1%) (Supplementary Table 3). In 2018, 114 of 190 healthcare workers self-reported seasonal influenza vaccination (60.0%). Self-reported influenza vaccination was not significantly different between survey years. Influenza vaccination coverage ranged from 46.2–80.0% between hospitals (Figure 2).

Unadjusted analyses of all participants demonstrated the odds of self-reported current influenza vaccination were 8.64 times higher for healthcare workers who self-reported vaccination in previous year (95% CI: 6.02–12.40), 1.46 times higher for those who believed the influenza vaccine was composed of inactivated viruses (95% CI: 1.01–2.11), and 51% less for those who believed the influenza vaccine may cause harm (95% CI: 0.36–0.67) (Table 4). Results stratified by survey year were similar, except that the unadjusted odds of current influenza vaccination for participants in 2018 were also 54% less for healthcare workers who believed everyone has the same risk of getting sick or dying from influenza (95% CI: 0.23–0.85) (Supplementary Table 4).

In the final model of all participants, the odds of self-reported current influenza vaccination were 8.13 times higher for healthcare workers who self-reported vaccination in previous year, adjusting for the belief influenza vaccine may cause harm (Table 4). Adjusting for vaccination in previous year, the odds of self-reported current influenza vaccination were 38% less for those who believed the influenza vaccine may cause harm. Hosmer-Lemeshow goodness-of-fit test demonstrated the model fit was adequate (*p* = 0.52). Tolerance values for independent variables were > 0.97. Final model results restricted to participants in 2017 were similar (Supplementary Table 4). In the final model for participants in 2018, influenza vaccination was positively associated with vaccination in previous year (aOR: 31.10; 95% CI: 12.62–76.65) and inversely associated with the belief everyone has the same risk of getting sick from influenza (aOR: 0.27; 95% CI: 0.11–0.66).

Of 393 healthcare workers who were vaccinated for influenza, 135 (34.4%) reported mild or moderate untoward reactions after vaccination, including vaccination site pain, flu-like symptoms, and general discomfort (Supplementary Table 5).

### Reasons for non-vaccination

Among 313 unvaccinated healthcare workers, the main reasons for non-vaccination were fear of side effects or developing disease (49.5%) and access limitations (e.g., time constraints, vaccine not offered) (26.5%) (Table 5). There were no significant differences in reasons for non-vaccination between survey years (Supplementary Table 6).

## Discussion

In this study of 747 healthcare workers of public hospitals in Costa Rica, seasonal influenza vaccine coverage was 54.1% in 2017 and 60.0% in 2018, lower than the coverage reported by PAHO for the same period (88%, 2017) and (72%, 2018) [19]. However, this study only included healthcare workers attending patients, whereas the PAHO figures also included administrative staff who were not in contact with patients. Vaccination coverage in this study fell short of the 80% vaccination rate threshold proposed to reach herd immunity within healthcare facilities for seasonal influenza [26]. Suboptimal seasonal influenza vaccination coverage among healthcare workers may be attributed to misunderstandings of influenza virus and vaccine, which is consistent with other studies of healthcare workers [14,15,27]. Although a trivalent inactivated influenza vaccine is used in Costa Rica during vaccination campaigns, four-fifths of healthcare workers believed the vaccine was composed of active viruses. Furthermore, nearly all participants believed vaccination causes flu-like symptoms, one-fifth of vaccinated healthcare workers reported flu-like symptoms after vaccination, half of unvaccinated participants cited fear of side effects or developing influenza as reasons for declining vaccination, and those who believed the vaccine may be harmful were less likely to be vaccinated than those who did not. Healthcare providers with negative views of vaccination have been shown to be less likely to recommend, or even discourage, vaccination among risk groups [28]. The most cited source of information about influenza vaccination was mass media, which has been demonstrated to be an influential factor in refusing influenza vaccination [29]. It is also conceivable that a heightened influenza season in Costa Rica in 2017 affected perceptions of vaccination efficacy [30]. Only one-quarter of participants cited workplace trainings as a source of information of vaccination, so routine workplace trainings are needed to help healthcare workers discern credible from non-credible sources of information.

Direct physician recommendations have been shown to be associated with seasonal influenza vaccination among risk groups, including pregnant women [31], older adults [32], and individuals with comorbidities [33]. One systematic review found that pregnant women who received recommendations from healthcare providers were 20 to 100 times more likely to be vaccinated [28]. Healthcare workers who themselves are vaccinated serve as the best advocates for seasonal influenza vaccination campaigns [34].

The finding that healthcare workers who believed everyone has the same risk of getting sick from influenza were less likely to have been vaccinated for influenza in 2018 is consistent with other studies [34,35]. This finding may suggest that healthcare workers did not believe themselves to be at higher risk of infection or that all patients have similar risk profiles. Healthcare workers with a higher perception of risk of infection were more likely to be vaccinated in other studies to prevent transmission to their patients [34]. Lack of time for vaccination accounted for 16% of responses among non-vaccinated participants, which is consistent with other studies [36, 37]. Perhaps local hospitals could set their own internal goal of 100% coverage for healthcare workers and monitor progress toward that goal throughout the influenza season. Other reasons for non-vaccination among healthcare workers reported in other studies including high vaccination costs, fear of needles, and low perception of risk [14,15] were not reasons disclosed for non-vaccination in this study. Mandatory vaccination policies for healthcare workers, with exemptions only for employees with medical contraindications, are generally well-accepted and may improve seasonal influenza vaccination rates [38,39]. Other strategies including education, on-site vaccinations, mobile vaccination carts, incentives, vaccine champions (e.g., individuals who advocate for use of recommended vaccinations), requiring unvaccinated healthcare workers to wear a mask, walk-in vaccinations, and requiring unvaccinated employees to sign declination forms, have had some success at improving influenza vaccination rates among healthcare workers [40,41].

Despite suboptimal influenza vaccination coverage, 96.1% of participants agreed healthcare personnel should be vaccinated for influenza annually and 86.5% would do so if the vaccine was easily accessible. Consistent with other studies, vaccination in the previous year had the strongest association with the current vaccination [32,42,43]. One review study found that healthcare workers in hospitals who had previously been vaccinated for influenza were 5.4 to 1,000 times more likely to have been vaccinated the following year [44]. Investing in an intense influenza vaccination campaign particularly aimed at young adults to foster mindsets conducive to lifelong annual vaccination as a matter of routine may increase coverage. There was no significant difference in influenza vaccination coverage between survey years, but a greater proportion of participants in 2018 knew vaccination is safe and effective, people may contract influenza multiple times, and the virus may be transmitted via direct contact with an infected individual. We surveyed healthcare workers at different hospitals between survey years, so these differences may be attributed to different medical practices, previous experiences with influenza, funding schemes, surveillance systems, vaccination attitudes, and promotional activities [45]. Demographics associated with seasonal influenza vaccination in other studies (older age, male sex, years in profession, married, physician profession) [12, 42] were not associated with vaccination in this study.

This study had several limitations. First, surveys were completed during two time periods to meet new requirements of the National Health Research Council of Costa Rica. As KAP of influenza and the environment may have differed between years, we reported overall results and results stratified by survey year, which had lower statistical power. Second, this study focused on healthcare workers attending patients in hospitals and may not be generalizable to other clinical settings. Third, this was a cross-sectional study, so causal inferences may not be drawn from results. Fourth, influenza vaccinations were self-reported, which may overestimate vaccination coverage, although other studies have shown strong concordance between self-reported influenza vaccination and medical records [46]. Fifth, there may have been recall and social desirability bias in self-reported KAP responses. Sixth, there may have been response bias if motivated and vaccinated healthcare workers were more likely to participate than unvaccinated individuals. Notwithstanding these limitations, our study included a large sample of healthcare workers from CCSS. To our knowledge, this is the first KAP study of seasonal influenza vaccination in Costa Rica. Seasonal influenza vaccination prevents healthcare workers from contracting influenza and spreading influenza to their patients. Influenza vaccination coverage among healthcare workers in a sample of Costa Rican hospitals was suboptimal due to knowledge gaps, misconceptions, and fears regarding vaccine safety. Vaccination in the previous year had the strongest association with current vaccination. Young adults in the workplace may be the best targets for information and vaccination campaigns to encourage a lifelong commitment to annual vaccination. There is a need for appropriate interventions to increase healthcare worker vaccination coverage and improve their knowledge and beneficence, which would improve patient safety in hospitals.

## Supplementary Material

1

## Figures and Tables

**Table 1.** Demographics and influenza vaccination coverage of 747 healthcare workers^a^, Costa Rica, 2017–2018.

Characteristic	n (%)
**Age (in years)**	
20–30	169 (22.6)
31–40	272 (36.4)
≥41	306 (41.0)
Female sex	445 (59.6)
**Marital status**	
Single	256 (34.3)
Married	391 (52.3)
Divorced	94 (12.6)
Other	6 (0.8)
**Profession**	
Doctor	196 (26.3)
Nursing professional	201 (26.9)
Nursing assistant	110 (14.7)
Other healthcare profession	240 (32.1)
**Years in profession**	
≤10	347 (46.4)
11–20	206 (27.6)
≥21	194 (26.0)
Works in multiple healthcare facilities	120 (16.1)
Self-reported influenza vaccination in previous year (n = 696)^b^	449 (64.5)
Self-reported current influenza vaccination (n=706)^b^	393 (55.7)

a See Supplementary Table 3 for results stratified by survey year;

b Excluded participants who did not know their vaccination status or did not respond.

**Table 2.** Knowledge and attitudes towards influenza vaccine, healthcare workers^a^, Costa Rica, 2017–2018.

Knowledge and attitudes	Participants^b^	Agree	
	n	n	% (95% CI)
**Knowledge of influenza**			
Influenza may be transmitted from person to person	746	708	94.9 (93.3–96.5)
Influenza may be transmitted from birds or pigs to people	745	461	61.9 (58.4–65.4)
People may contract influenza even if they have previously contracted influenza	747	675	90.4 (88.2–92.5)
Influenza may be transmitted via droplets from coughs or sneezes	747	732	98.0 (97.0–99.0)
Influenza may be transmitted if people touch their mouths or noses with contaminated hands	747	634	85.0 (82.4–87.6)
Everyone has the same risk of getting sick or dying from influenza	747	213	28.5 (25.3–31.8)
The vaccine protects against influenza complications	741	710	95.8 (94.4–97.3)
The influenza vaccine is composed of inactivated viruses	747	155	20.7 (17.8–23.7)
**Attitudes towards the influenza vaccine**			
The influenza vaccine may cause harm	703	252	35.8 (32.3–39.4)
Healthcare personnel should get vaccinated for influenza every year	700	673	96.1 (94.7–97.6)
The influenza vaccine causes flu-like symptoms	708	670	94.6 (93.0–96.3)
Would get vaccinated for influenza if offered the vaccine at work	703	608	86.5 (84.0–89.0)
Recommends the influenza vaccine to family and friends	698	625	89.5 (87.3–91.8)

CI: confidence interval;

a See Supplementary Table 1 for results stratified by survey year;

b Excluded healthcare workers who did not respond.

**Table 3.** Sources of information about influenza vaccination, healthcare workers^a^, Costa Rica, 2017–2018.

Source of information	All participants (N = 747)	
	N	% (95% CI)
Conversations with family	71	9.5 (7.4–11.6)
Conversations with friends or coworkers	284	38.0 (34.5–41.5)
Mass media	466	62.4 (58.9–65.9)
Informal information from the healthcare facility	439	58.8 (55.2–62.3)
Training in the healthcare facility	202	27.0 (23.8–30.2)
Doctor or nurse at healthcare facility	282	37.8 (34.3–41.2)
Medical consultation	95	12.7 (10.3–15.1)

CI: confidence interval;

a See Supplementary Table 2 for results stratified by survey year.

**Table 4.** Associations between demographics, sources of information, knowledge and attitudes, and influenza vaccination, healthcare workers^a^, Costa Rica, 2017–2018 (N = 688)^b^.

Variable	Vaccinated n (%)	OR (95% CI)	aOR^c^ (95% CI)
Age in years (Ref: 20–30)			
31–40	134 (52.3)	0.92 (0.61–1.37)	–
≥41	165 (59.4)	1.22 (0.82–1.81)	–
Male sex (Ref: female)	156 (56.1)	1.03 (0.76–1.40)	–
Marital status (Ref: single)			
Married	204 (56.5)	1.07 (0.77–1.49)	-
Divorced	48 (54.6)	0.99 (0.60–1.61)	–
Other	2 (50.0)	0.82 (0.12–5.93)	–
Profession (Ref: doctor)			
Nursing professional	101 (54.9)	1.27 (0.84–1.91)	–
Nursing assistant	56 (56.6)	1.36 (0.83–2.22)	–
Other	135 (60.6)	1.54 (0.92–2.46)	–
Years in profession (Ref: ≤ 10)			
11–20	106 (55.5)	1.05 (0.74–1.51)	–
≥21	103 (58.5)	1.19 (0.82–1.73)	–
Works in multiple healthcare facilities (Ref: no)	55 (48.3)	0.70 (0.47–1.05)	–
Source of information (Ref: not a source of information)			
Conversations with family	44 (65.7)	1.59 (0.94–2.70)	–
Conversations with friends or coworkers	150 (55.0)	0.95 (0.70–1.30)	–
Mass media	245 (54.0)	0.82 (0.59–1.12)	–
Informal information from the healthcare facility	240 (56.1)	1.05 (0.77–1.42)	–
Training in the healthcare facility	117 (59.4)	1.24 (0.89–1.73)	–
Doctor or nurse at healthcare facility	158 (57.0)	1.10 (0.81–1.49)	–
Medical consultation	55 (59.1)	1.18 (0.76–1.84)	–
Knowledge and attitudes (Ref: no)			
Believe influenza may be transmitted from person to person	362 (55.2)	0.65 (0.31–1.36)	–
Believe influenza may be transmitted from birds or pigs to people	226 (53.4)	0.79 (0.58–1.08)	–
Believe people may contract influenza multiple times	349 (55.9)	1.12 (0.67–1.88)	–
Believe influenza may be via droplets from coughs or sneezes	374 (55.4)	0.55 (0.17–1.81)	–
Believe influenza may be transmitted if people touch their mouths or noses with contaminated hands	330 (56.5)	1.25 (0.82–1.90)	–
Believe everyone has the same risk of getting sick or dying from influenza	105 (53.9)	0.90 (0.65–1.26)	–
Believe the influenza vaccine is composed of inactivated viruses	96 (62.8)	1.46 (1.01–2.11)	–
Believe the influenza vaccine may cause harm	110 (44.4)	0.49 (0.36–0.67)	0.62 (0.44–0.89)
Believe the influenza vaccine causes flu-like symptoms	358 (54.9)	0.54 (0.26–1.11)	–
Vaccinated for influenza in previous year (Ref: no)	325 (73.0)	8.64 (6.02–12.40)	8.13 (5.65–11.71)

Ref: reference; OR: odds ratio; aOR: adjusted odds ratio; CI: confidence interval;

a See Supplementary Table 4 for results stratified by survey year;

b Analyses excluded individuals who did not know or did not provide their vaccination status and those who did respond to questions regarding knowledge and attitude of influenza vaccination;

c Adjusted for the other variables listed in the model.

**Table 5.** Reasons for not receiving influenza vaccination, healthcare workers (N = 313)^a^, Costa Rica, 2017–2018.

Reasons	n	% (95% CI)
*Limited access*	83	26.5 (21.6–31.4)
Did not have time to get vaccinated	51	16.3 (12.2–20.4)
Vaccine was not offered	38	12.1 (8.5–15.8)
Did not know where to go for vaccine	8	2.6 (0.8–4.3)
*Rejection*	210	67.1 (61.9–72.3)
Fear of side effects	128	40.9 (35.4–46.4)
Fear of contracting influenza	95	30.4 (25.2–35.5)
Believed influenza does not cause serious illness	41	13.1 (9.3–16.9)
Believed vaccine is ineffective	31	9.9 (6.6–13.2)
Was sick	24	7.7 (4.7–10.6)

CI: confidence interval; Composite subheadings (limited access, rejection) included at least one positive response for one of the listed reasons;

a See Supplementary Table 6 for results stratified by survey year.
