# Supplementary material for: Knowledge, attitudes, and practices for the use of seasonal influenza vaccination, healthcare workers, Costa Rica
Source: J Infect Dev Ctries. Author manuscript; Available in PMC 2022 May 25. (PMC9131022; doi:10.3855/jidc.14381)
Supplement: 1 [file NIHMS1809015-supplement-1.pdf]

Annex – Supplementary Items

Supplementary Table 1. Knowledge and attitudes of influenza virus, transmission, and vaccine stratified by survey year, healthcare workers, Costa Rica, 2017-2018.

Knowledge and attitudes	2017			2018			p-value[b]
	Total[a]	Agree		Total[a]	Agree		
	N	N	% (95% CI)	n	n	% (95% CI)	
**Knowledge of influenza**							
Influenza may be transmitted from person to person	553	521	94.2 (92.3–96.2)	193	187	96.9 (94.4–99.4)	0.15
Influenza may be transmitted from birds or pigs to people	551	347	63.0 (58.9–67.0)	194	114	58.8 (51.8–65.7)	0.30
People may contract influenza multiple times	553	490	88.6 (86.0–91.3)	194	185	95.4 (92.4–98.3)	0.01
Influenza may be transmitted via droplets from coughs or sneezes	553	544	98.4 (97.3–99.4)	194	188	96.9 (94.5–99.3)	0.21
Influenza may be transmitted if people touch their mouths or noses with contaminated hands	553	460	83.2 (80.1–86.3)	193	174	90.2 (85.9–94.4)	0.02
Everyone has the same risk of getting sick or dying from influenza	553	160	28.9 (25.1–32.7)	194	53	27.3 (21.0–33.6)	0.67
The vaccine protects against influenza complications	548	520	94.9 (93.0–96.7)	193	190	98.4 (96.7–100)	0.03
The influenza vaccine is composed of inactivated viruses	553	118	21.3 (17.9–24.8)	194	37	19.1 (13.5–24.6)	0.50
**Attitudes towards the influenza vaccine**							
The influenza vaccine may cause harm	517	201	38.9 (34.7–43.1)	186	51	27.4 (20.9–33.9)	0.01
Healthcare personnel should get vaccinated for influenza every year	512	488	95.3 (93.5–97.1)	188	185	98.4 (96.6–100)	0.06
The influenza vaccine causes flu-like symptoms	518	489	94.4 (92.4–96.4)	190	181	95.3 (92.2–98.3)	0.65
Would get vaccinated for influenza if offered the vaccine at work	514	436	84.8 (81.7–87.9)	189	172	91.0 (86.9–95.1)	0.03
Recommends the influenza vaccine to family and friends	511	454	88.8 (86.1–91.6)	187	171	91.4 (87.4–95.5)	0.32

CI: confidence interval; [a] Excluded healthcare workers who did not respond; [b] P-value from Pearson Chi-square test.

Supplementary Table 2. Sources of information about influenza vaccination stratified by survey year, healthcare workers, Costa Rica, 2017-2018.

Source of information	2017 (n = 553)		2018 (n = 194)		p-value[a]
	N	% (95% CI)	N	% (95% CI)	
Conversations with family	62	11.2 (8.6–13.8)	9	4.6 (1.7–7.6)	< 0.01
Conversations with friends or coworkers	247	44.7 (40.5–48.8)	37	19.1 (13.5–24.6)	< 0.01
Mass media	360	65.1 (61.1–69.1)	106	54.6 (47.6–61.7)	0.01
Informal information from the healthcare facility	331	59.9 (55.8–64.0)	108	55.7 (48.6–62.7)	0.31
Training in the healthcare facility	131	23.7 (20.1–27.2)	71	36.6 (29.8–43.4)	< 0.01
Doctor or nurse at healthcare facility	207	37.4 (33.4–41.5)	75	38.7 (31.7–45.6)	0.76
Medical consultation	78	14.1 (11.2–17.0)	17	8.8 (4.7–12.8)	0.05

CI: confidence interval; [a] P-value from Pearson Chi-square test.

Supplementary Table 3. Demographics and influenza vaccination coverage of 747 healthcare workers stratified by survey year, Costa Rica, 2017-2018.

Characteristic	2017<br>N = 553<br>N (%)	2018<br>N = 194<br>N (%)	p-value[a]
**Age (in years)**			**0.21**
20-30	134 (24.2)	35 (18.0)	
31-40	197 (35.6)	75 (38.7)	
≥41	222 (40.2)	84 (43.3)	
Female sex	319 (57.7)	126 (65.0)	0.08
**Marital status**			**0.16**
Single	188 (34.0)	68 (35.0)	
Married	296 (53.5)	95 (49.0)	
Divorced	63 (11.4)	31 (16.0)	
Other	6 (1.1)	0 (0)	
**Profession**			**< 0.01**
Doctor	153 (27.6)	43 (22.2)	
Nursing professional	141 (25.5)	60 (30.9)	
Nursing assistant	101 (18.3)	9 (4.6)	
Other healthcare profession	158 (28.6)	82 (42.3)	
**Years in profession**			**0.24**
≤10	267 (48.3)	80 (41.2)	
11-20	148 (26.8)	58 (29.9)	
≥21	138 (24.9)	56 (28.9)	
Works in multiple healthcare facilities	95 (17.2)	25 (12.9)	0.16
Self-reported influenza vaccination in previous year[b]	324 (63.5)	125 (67.2)	0.37
Self-reported current influenza vaccination[c]	279 (54.1)	114 (60.0)	0.16

[a] P-value from Pearson Chi-square test; [b] Self-reported influenza vaccination in previous year (2017: n = 510; 2018: n = 186). Excluded participants who did not know their vaccination status or did not respond; [c] Self-reported influenza vaccination (2017: n = 516; 2018: n = 190). Excluded participants who did not know their vaccination status or did not respond.

Supplementary Table 4. Associations between demographics, sources of information, knowledge and attitudes, and influenza vaccination stratified by survey year, healthcare workers, Costa Rica, 2017-2018.

Variable	2017 (N = 505)[a]		2018 (N = 183)[a]	
	OR (95% CI)	aOR[b] (95% CI)	OR (95% CI)	aOR[b] (95% CI)
Age in years (Ref: 20-30)				
31-40	0.78 (0.49–1.24)	–	1.46 (0.64–3.35)	–
≥ 41	1.03 (0.65–1.63)	–	1.96 (0.87–4.44)	–
Male sex (Ref: female)	1.13 (0.79–1.61)	–	0.83 (0.45–1.55)	–
Marital status (Ref: single)				
Married	0.98 (0.67–1.44)	–	1.41 (0.73–2.70)	–
Divorced	0.77 (0.42–1.39)	–	1.66 (0.67–4.10)	–
Other	0.82 (0.11–5.95)	–	–	–
Profession (Ref: doctor)				
Nursing professional	1.47 (0.91–2.38)	–	0.76 (0.34–1.70)	–
Nursing assistant	1.51 (0.89–2.56)	–	1.54 (0.27–8.89)	–
Other	1.54 (0.92–2.46)	–	1.06 (0.49–2.30)	–
Years in profession (Ref: ≤10)				
11-20	0.83 (0.55–1.27)	–	2.00 (0.97–4.14)	–
≥21	0.97 (0.63–1.51)	–	2.00 (0.97–4.14)	–
Works in multiple healthcare facilities (Ref: no)	0.68 (0.43–1.08)	–	0.80 (0.34–1.88)	–
Source of information (Ref: not a source of information)				
Conversations with family	1.63 (0.93–2.88)	–	2.00 (0.39–10.19)	–
Conversations with friends or coworkers	1.06 (0.74–1.50)	–	0.86 (0.40–1.84)	–
Mass media	0.91 (0.62–1.33)	–	0.70 (0.38–1.27)	–
Informal information from the healthcare facility	0.70 (0.38–1.27)	–	1.07 (0.75–1.54)	–
Training in the healthcare facility	1.47 (0.98–2.21)	–	0.76 (0.41–1.40)	–
Doctor or nurse at healthcare facility	1.20 (0.84–1.72)	–	0.85 (0.47–1.56)	–
Medical consultation	1.57 (0.95–2.60)	–	0.42 (0.15–1.15)	–
Knowledge and attitudes (Ref: no)				
Believe influenza may be transmitted from person to person	0.72 (0.32–1.61)	–	0.30 (0.03–2.61)	–
Believe influenza may be transmitted from birds or pigs to people	0.80 (0.56–1.15)	–	0.79 (0.43–1.45)	–
Believe people may contract influenza multiple times	1.10 (0.63–1.92)	–	0.92 (0.21–3.98)	–
Believe influenza may be via droplets from coughs or sneezes	0.38 (0.08–1.92)	–	1.03 (0.17–6.31)	–
Believe influenza may be transmitted if people touch their mouths or noses with contaminated hands	1.31 (0.82–2.09)	–	0.89 (0.33–2.38)	–
Believe everyone has the same risk of getting sick or dying from influenza	1.17 (0.79–1.72)	–	0.44 (0.23–0.85)	0.27 (0.11–0.66)
Believe the influenza vaccine is composed of inactivated viruses	1.31 (0.86–1.99)	–	2.25 (0.99–5.12)	–
Believe the influenza vaccine may cause harm	0.53 (0.37–0.76)	0.63 (0.42–0.94)	0.41 (0.21–0.79)	–
Believe the influenza vaccine causes flu-like symptoms	0.45 (0.19–1.04)	–	0.92 (0.21–3.98)	–
Vaccinated for influenza in previous year (Ref: no)	6.27 (4.19–9.39)	5.96 (3.97–8.95)	25.75 (11.05–60.02)	31.10 (12.62–76.65)

Ref: reference; OR: odds ratio; aOR: adjusted odds ratio; CI: confidence interval; [a] Analyses excluded people who did not know or did not provide their vaccination status and those who did respond to questions regarding knowledge and attitude of influenza vaccination; [b] Adjusted for the other variables listed in the model.

Supplementary Table 5. Clinical manifestations seven days after vaccination, healthcare workers, Costa Rica, 2017-2018.

Clinical manifestation	All participants (n = 393)		2017 (n = 279)		2018 (n = 114)		p-value[a]
	n	% (95% CI)	n	% (95% CI)	n	% (95% CI)	
General discomfort	58	14.8 (11.2-18.3)	44	15.8 (11.5–20.1)	14	12.3 (6.2–18.4)	0.38
Vaccination site pain	76	19.3 (15.4-23.3)	54	19.4 (14.7–24.0)	22	19.3 (11.9–26.7)	0.99
Fever	31	7.9 (5.2-10.6)	21	7.5 (4.4–10.6)	10	8.8 (3.5–14.0)	0.68
Flu-like symptoms	80	20.4 (16.4-24.4)	61	21.9 (17.0–26.7)	19	16.7 (9.7–23.6)	0.25
Headache	9	2.3 (0.8-3.8)	6	2.2 (0.4–3.9)	3	2.6 (0–5.6)	0.77

CI: confidence interval; [a] P-value from Pearson Chi-square test.

Supplementary Table 6. Reasons for not receiving influenza vaccination stratified by survey year, healthcare workers, Costa Rica, 2017-2018.

Reasons	2017 (N = 237)		2018 (N = 76)		p-value[a]
	n	% (95% CI)	n	% (95% CI)	
Limited access	66	27.8 (22.1–33.6)	17	22.4 (12.8–32.0)	0.35
Did not have time to get vaccinated	41	17.3 (12.4–22.2)	10	13.2 (5.4–20.9)	0.39
Vaccine was not offered	30	12.7 (8.4–16.9)	8	10.5 (3.5–17.6)	0.62
Did not know where to go for vaccine	7	3.0 (0.8–5.1)	1	1.3 (0–3.9)	0.43
Rejection	160	67.5 (61.5–73.5)	50	65.8 (54.9–76.7)	0.78
Fear of side effects	99	41.8 (35.4–48.1)	29	38.2 (27.0–49.3)	0.58
Fear of contracting influenza	78	32.9 (26.9–38.9)	17	22.4 (12.8–32.0)	0.08
Believed influenza does not cause serious illness	33	13.9 (9.5–18.4)	8	10.5 (3.5–17.6)	0.44
Believed vaccine is ineffective	26	11.0 (7.0–15.0)	5	6.6 (0.9–12.3)	0.26
Was sick	20	8.4 (4.9–12.0)	4	5.3 (0.1–10.4)	0.37

CI: confidence interval; Composite subheadings (limited access, rejection) included at least one positive response for one of the listed reasons; [a] P-value from Pearson Chi-square test.

Supplementary File 1. Sample size formula

The following equation was used to obtain sample sizes for surveys of healthcare workers (Schaeffer R, Mendenhall W, Ott L [1990] Elementary Survey Sampling, Boston. Massachusetts: PWS-Kent Publishing Company):

$$n = deff \times \frac{NZ_{1-\alpha/2}^2\, P(1-P)}{(N-1)d^2 + Z_{1-\alpha/2}^2\, P(1-P)}$$

Where:

N = population size

$Z_{1-\alpha/2}$ = Quantile of a variable with standard normal distribution

$100(1-\alpha)\%$ = Confidence level

P = Expected proportion in the population.

d = Absolute accuracy required

$deff$ = Design effect
